# A Comparative Study of Luteinizing Hormone Levels in Polycystic Ovarian Syndrome With Hyperandrogenism: Metformin Versus Oral Contraceptive Pills

**DOI:** 10.7759/cureus.29487

**Published:** 2022-09-23

**Authors:** Abdul Fattah, Dania A Al-Kader, Emilia E Jones Amaowei, Humayoun Amini, Hewad Hewadmal, Sayed Farhad Rasuli, Ijeoma V Ikedum, Jawad Farooq, Masharib Bashar, Laila Tul Qadar

**Affiliations:** 1 Internal Medicine, Indus Medical College, Tando Muhammad Khan, PAK; 2 Radiology, Liaquatian Research Council, Hyderabad, PAK; 3 Clinical Research, Dexcom, Inc., San Diego, USA; 4 Endocrinology, Liaquatian Research Council, Hyderabad, PAK; 5 Cardiology, Liaquatian Research Council, Hyderabad, PAK; 6 Cardiology, Sheikh Zayed Medical College/Hospital, Rahim yar Khan, PAK; 7 General and Colorectal Surgery, Liaquatian Research Council, Hyderabad, PAK; 8 Internal Medicine, Liaquatian Research Council, Hyderabad, PAK; 9 Medicine, Liaquat University of Medical and Health Sciences, Jamshoro, PAK; 10 Internal Medicine, Dr. Ruth KM Pfau Civil Hospital, Karachi, PAK; 11 Internal Medicine, Dow University of Health Sciences, Civil Hospital Karachi, Karachi, PAK

**Keywords:** menstrual irregularity, combined oral contraceptive pills, metformin, hyperandrogenism, pcos

## Abstract

Introduction

The primary objective of the study was to compare the serum luteinizing hormone (LH) levels in patients with hyperandrogenism on metformin and combined oral contraceptive pills. Secondarily, the study also assessed the serum testosterone, body mass index (BMI), and the time to achieve regular menstruation were also assessed.

Methods

A quasi-experimental study was conducted at the Department of Medicine, Liaquat University of Medical & Health Sciences (LUMHS) between June 1, 2019 and May 30, 2020. A total of 200 women fulfilling the clinical and biochemical criteria for the polycystic ovarian syndrome (PCOS) were enrolled, 100 in each group. Considering the inclusion criteria, the patients were picked up from the gynecology outpatient department. After taking a detailed history and physical, abdominal, and pelvic examination, pelvic ultrasonography along with biochemical evaluations of serum LH and testosterone were done in selected patients. Metformin was started at an oral dose of 500 mg daily and maintained at 1500 mg for six months in group A, and oral contraceptive pills were given for a period of six months in group B. Besides body weight and hirsutism, serum LH levels, serum prolactin levels, and serum testosterone levels were performed at the start of the treatment and then repeated after three months and after six months. After six months of menstrual cyclicity, changes in serum LH levels and body weights were assessed in the two groups and the rate of conception in the Metformin group.

Results

A total of 200 women were enrolled and equally divided into metformin and oral contraceptive groups. Follow-up revealed that a significantly higher number of patients achieved regular menstruation in the metformin group as compared to the oral contraceptive groups (p = 0.03). In the metformin group, 72 patients achieved regular menses, while in the oral contraceptive groups, about 58 patients achieved regular menstruation. Both metformin and oral contraceptive therapy were effective in improving patient outcomes in terms of serum LH, testosterone levels, and BMI. However, metformin had considerably higher rates of improvement as compared to oral contraceptive group patients. The mean serum LH level decreased from 38 mIU/ml to 17.6 mIU/ml in the metformin group (p < 0.0001), while the mean serum LH level reduced from 37.5 mIU/ml to 27.7 mIU/ml in the oral contraceptive group (p < 0.01). The change in serum testosterone level after six months was 2.98 ± 0.75 in the metformin group (p < 0.001) and 1.50 ± 0.64 in the oral contraceptive group (p < 0.01).

Conclusion

We revealed that both metformin and oral contraceptives are effective in improving symptomatology in PCOS patients. However, a significantly higher number of patients achieved normal menses with metformin than with oral contraceptives. Moreover, metformin had considerably higher rates of improvements in serum LH levels and serum testosterone levels as compared to oral contraceptive group patients.

## Introduction

Patients with polycystic ovarian syndrome (PCOS) may display symptoms like irregular menstrual cycles (from oligomenorrhea to amenorrhea), infertility, hirsutism, and obesity, and is a very prevalent endocrine condition [1]. The diagnostic criteria include hyperandrogenism and chronic anovulation (erratic or absent menstrual cycles) and the exclusion of other etiologies such as adrenal and ovarian neoplasms, hyperprolactinemia, congenital adrenal hyperplasia in adults, and malignancies that produce androgens. Recent definitions of PCOS require two of three symptoms: oligomenorrhea, anovulation, and hyperandrogenism [2].

PCOS is characterized biochemically by insulin resistance as well as compensatory hyperinsulinemia. At least 40% of females with PCOS have insulin resistance, indicating an association between insulin metabolism disturbances and PCOS [3]. Hyperinsulinemia induces the hyperandrogenism of PCOS by increasing ovarian androgen synthesis (especially testosterone and androstenedione) and decreasing sex hormone binding globulin (SHBG) buildup (a critical driver of the free androgen index). Increased levels of androgen hormones are associated with anovulation, amenorrhea, and infertility because they disrupt the pituitary-ovarian axis [4]. Furthermore, hyperinsulinemia has been linked to hypertension and hypercoagulability (due to elevated PAI-1 activity), and it appears to be a significant risk factor for the onset of chronic illnesses, including type II diabetes mellitus, heart disease, stroke, dyslipidemia, endometrial cancer, and breast cancer [4].

A family history of PCOS is common, and different symptoms may be inherited in different ways. It is possible for polycystic ovaries to exist without any outward manifestations of the syndrome but for symptoms to manifest later on, perhaps as a result of a lifestyle change like gaining weight. Multiple interconnected factors influence PCOS manifestation. The endocrine and metabolic profiles as well as symptoms are both negatively affected by weight gain, while they improve with weight loss [5]. Women of childbearing age often struggle with infertility, and PCOS is a major contributor to this problem (about 60%) [6-8].

The majority of patients (50-70%) attending outpatient departments are found to have PCOS, presenting with menstrual irregularity, weight gain, hirsutism, and infertility [7]. Convincing evidence suggests that leading medicines used to treat diabetes mellitus in adults, such as metformin, can reverse this endocrine abnormality. Liver glucose synthesis is decreased and insulin sensitivity is enhanced by this biguanide through reducing insulin secretion [7-10].

Hyperandrogenism is a critical characteristic of females with PCOS, and increased serum luteinizing hormone (LH) results in excess production of androgens. The present study aimed to assess the reduction in serum LH levels in patients with hyperandrogenism who are prescribed metformin as compared to those who are prescribed combined oral contraceptive pills.

## Materials and methods

A quasi-experimental study was conducted at the Department of Medicine, Liaquat University of Medical and Health Sciences (LUMHS) between June 1, 2019, and 30th May 2020. Participants were enlisted using a convenience sample method, which is not based on probability. The research project started only after getting official approval from the Liaquat University of Medical and Health Sciences ethical review board (IRB# IRB/139/2019).

All women with an age above 18 years and diagnosed with the PCOS who received treatment at our center were included in the study. According to the type of care provided, the entire patient population was split in half. Individuals in group A were offered combined oral contraceptive pills, while the second group was given metformin. Women with hyperprolactinemia, adult-onset congenital adrenal hyperplasia, androgen-producing neoplasms, Cushing syndrome, and underlying thyroid dysfunction were excluded from this research.

Informed consent was obtained from each participant after the objectives of the study were narrated to them. A detailed history of the patient was taken regarding menstrual, obstetric, and family histories. A physical examination was performed upon presentation, and an abdominal examination was carried out for any palpable swelling. All relevant investigations, like ultrasonography and a hormonal profile, were performed. On ultrasonography, polycystic ovaries were identified as having several peripheral cysts (5-9 mm) and a follicle-stimulating hormone (FSH) to LH ratio proportion of greater than 1:3. The severity of hirsutism was documented using the Ferriman Gallwey score. The upper lip, chin, chest, back, arms, abdominal and pelvic regions were examined for hair growth. Higher scores indicated severe hirsutism.

Weights in kilograms were determined using a weight scale at presentation and before initiating the treatment and then rechecked after the completion of the six-month treatment. One group received metformin for six months. Metformin was prescribed to be taken as follows: once daily for the first week; twice daily for the second week; and three times daily (1500 mg) for the final four weeks and the final six months.

Combined oral contraceptive pills (Levonorgestrel 0.15 mg and Ethinylestradiol 0.03 mg) were prescribed to the second group for the same time period. Serum LH levels, serum prolactin levels, and serum testosterone levels were recorded at the start of the treatment, rechecked after three months, and then after six months in both groups. At six months, regulation of menstrual periods, change of serum LH levels, and weight reduction were assessed in both groups. All data were documented on a designed proforma. The patients were said to have attained a regular menstrual cycle if the duration ranged between 21 and 40 days.

Data were entered into Statistical Package for Social Sciences (SPSS version 10, IBM Corp., Armonk, NY). Quantitative data, including variables for age of women, weight in kilograms, serum LH levels, serum testosterone levels, serum progesterone levels, and time in months, were analyzed by a t-test. Qualitative data, including hirsutism were analyzed by the chi-square test. A student t-test was used to assess the difference between the variables. Statistical significance was defined as p ≤ 0.05.

## Results

A total of 200 women were enrolled in the study, with a mean age of 26.5 ± 5.6 years. Women were equally divided into the metformin group (group A) and the oral contraceptives group (group B). Participants in both groups shared similar demographics, as demonstrated in Table 1.

**Table 1:** Patient demographics of the two groups.

Parameters	Metformin (n=100)	Standard (n=100)	p-value
Age (years)	26.68 ± 6.1	26.32 ± 5.1	0.143
Total LH levels, mIU/ml	38.0 ± 7.99	37.5 ± 8.2	0.41
Total testosterone, ng/dL	56.4 ± 21.8	54.1 ± 19.4	0.646
Body mass index (kg/m^2^)	34.25 ± 3.6	33.44 ± 3.5	0.363
Hirsutism			0.398
Mild	31 (31%)	29 (29%)	
Moderate	24 (24%)	22 (22%)	

Table 2 highlights the time it takes patients to achieve normality in menstrual flow. It was found that there was no discernable difference between the metformin and contraceptive groups in terms of achieving regularity of menstruation (p = 0.213). Overall, a significantly higher number of patients achieved regular menstruation in the metformin group as compared to the oral contraceptive groups (p = 0.03). In the metformin group, 72 patients achieved regular menses, while in oral contraceptive groups, about 58 patients achieved regular menstruation (Table 2).

**Table 2:** Distribution of patients time to get regular menstruation.

Time to get regular menstruation	Metformin group	Standard group	P-value
Normality was not achieved	28 (28%)	42 (42%)	0.213
Upto three months	24 (24%)	18 (18%)	
3-6 months	32 (32%)	28 (28%)	
More than six months	16 (16%)	12 (12%)	

Both metformin and oral contraceptive therapy were effective in improving patient outcomes in terms of serum LH, testosterone levels, and body mass index (BMI). However, metformin had considerably higher rates of improvement as compared to oral contraceptive group patients. The mean serum LH level decreased from 38 mIU/ml to 17.6 mIU/ml in the metformin group (p < 0.0001), while the mean serum LH level reduced from 37.5 mIU/ml to 27.7 mIU/ml in the oral contraceptive group (p < 0.01). The change in serum testosterone level after six months was 2.98 ± 0.75 in metformin group (p < 0.001) and 1.50 ± 0.64 (p < 0.01) in the oral contraceptive group (p < 0.01). Similar patterns were observed in BMI (Table 3).

**Table 3:** Decrease in variables analyzed by paired t-test between the two groups.

Group variable	Metformin (n=100)	p-value	Standard (n=100)	p-value
Serum LH levels (mIU/ml)	20.4 ± 8.24	<0.001	9.8 ± 6.5	<0.01
Serum testosterone levels (ng/dL)	2.98 ± 0.75	<0.001	1.50 ± 0.64	<0.001
Change in body mass Index (kg/m^2^)	6.7 ± 3.01	<0.001	2.0 ± 0.45	<0.01

## Discussion

The present study assessed the changes in serum LH and other parameters in individuals with polycystic ovary syndrome (PCOS) who are administered metformin as compared to those who are prescribed oral contraceptives. A discernible change in serum levels among the two groups was observed, and both were significantly effective in decreasing the serum LH, serum testosterone, and BMI of individuals suffering from PCOS.

As of now, there is no agreement on whether or not metformin should be used as first-line therapy in all patients with PCOS in an effort to induce ovulation, or if the drug metformin should be attempted after clomiphene citrate has ceased to induce ovulation. It is reasonable to use metformin as a first-line treatment for inducing ovulation in accordance with the most up-to-date version of the Cochrane database of systematic reviews concerning the use of insulin-sensitizing agents in individuals with PCOS [11]. Metformin use has been shown to reduce fasting insulin levels and low-density lipoprotein, along with hypertension in PCOS [11].

However, whether or not these modifications translate into a reduced risk of cardiovascular disease and diabetes is unclear in the absence of long-term data [12]. As a result, practitioners often prescribe additional pharmacologic interventions tailored to metabolic abnormalities. In human studies [13], metformin has been shown to reduce hirsutism, stimulate ovulation, and restore a regular menstrual cycle. One study of 39 females with PCOS and hyperinsulinemia found that metformin administration improved clinical features of hyperandrogenism and menstrual cycles by significantly lowering insulin and total and free testosterone [14]. Studies show that losing weight through exercise and dietary changes alone is just as effective as hormonal therapy for regulating menstrual periods and improving hyperandrogenism [15,16].

Metformin helps women with PCOS lose weight, and it works even better when combined with a healthy diet and regular exercise. Reducing insulin levels and the risk of developing diabetes can be accomplished by modifying one's eating habits and level of exercise, along with Metformin [12]. The present study revealed that patients who were on Metformin had a mean decrease in BMI of 6.7 ± 3.01 kg/m2.

A daily dose of metformin can range from 1,500 mg to 2,000 mg. Its primary adverse effects are digestive system-related and include gastrointestinal symptoms like sickness, throwing up diarrhoea, and farting. These conditions typically only last for a short time. These unwanted effects might be lessened by taking a higher dose gradually. Side effects are also decreased when taken with food. Rosiglitazone and pioglitazone are two additional insulin-sensitizing agents that can be used today.

The mean age of patients in this study was approximately 26 years, which is in line with the results of other studies [12-16]. As this endocrinological problem is common among women of reproductive age, early diagnosis and management are useful to prevent both the short-term as well as long-term sequelae of the disease.

Patients diagnosed with PCOS commonly exhibit the clinical feature of weight gain. Women with a BMI equal to 30 or higher must be encouraged to lose weight because their symptoms and endocrine profile will improve considerably with weight loss. Lifestyle modification is very important in treating PCOS, as weight loss and exercise show a striking improvement in ovulatory functions and features of hyperandrogenism. In this study, the average BMI change in the metformin group was 6.7 ± 3.01 kg/m2. According to a study, six months into Metformin therapy, 36 women (72%), and six months into oral contraceptive pill treatment, 29 women (58%) established menstrual cyclicity [17]. These results are similar to those of the current study.

There were a few limitations that merit mentioning. The first of these was a small sample size. Another factor is how much the participant understands about the therapy, so counseling patients about therapy is very important. Last, but perhaps not least, was the sampling technique. Because of the shortage of time and consequently the number of patients, we selected our patients by simple random sampling. This non-probability sampling is clearly a source of bias and less authenticity. Results based on such biases can, therefore, not be generalized.

## Conclusions

The conclusion of the study is that metformin treatment in patients with polycystic ovary syndrome may be a useful option for correcting menstrual irregularity in most women and results in considerable ovulation rates. Both metformin and oral contraceptive therapy were effective in improving patient outcomes in terms of serum LH, testosterone levels, and BMI. However, metformin had considerably higher rates of improvement as compared to oral contraceptive group patients.
